# Extended use of extra corporeal membrane oxygenation as bridge to lung transplantation in two patients

**DOI:** 10.1186/s13019-020-1046-0

**Published:** 2020-01-13

**Authors:** Elin Skansebo, Michael Broomé, Jesper Magnusson, Gerdt C. Riise, Göran Dellgren

**Affiliations:** 1 Transplant Institute, Sahlgrenska University Hospital, Sahlgrenska Academy, University of Gothenburg, Gothenburg, Sweden; 2 ECMO-centre, Karolinska University Hospital, Stockholm, Sweden; 3 Department Cardiothoracic Surgery, Sahlgrenska University Hospital, Sahlgrenska Academy, University of Gothenburg, SE-, 413 45 Gothenburg, Sweden

**Keywords:** Lung transplantation, ECMO, Mechanical circulatory support

## Abstract

**Background:**

We have previously reported our outcome after extra-corporeal membrane oxygenation as bridge-to-lung transplantation, which initially was considered controversial, but over time have gained acceptance and now is performed in most high-volume institutions.

**Case presentation:**

We now report two “extreme” extra-corporeal membrane oxygenation (ECMO) bridge-to-lung transplantation cases, on ECMO > 200 days prior to lung transplantation. One patient survived long-term and the other one did not, and clinical cause and morbidity is outlined in this case-report.

**Conclusion:**

We believe these two cases highlight the medical, ethical and resource allocation difficulties involved with saving patients in very dire circumstances. We have shown that a patient can survive extremely long duration of ECMO bridge to lung transplantation, but selection remains crucial to achieve a reasonable cost-benefit.

## Background

We and others have reported outcome after extra-corporeal membrane oxygenation (ECMO) bridge to lung transplantation (LTx) (1, 2). In many of these reports median waiting time to LTx is relatively short, in our recent cohort it was 9 days for a mean, since these patients get urgency priority on the waiting list in most organ allocation systems. In this case report we describe the outcome in 2 patients after considerably longer duration of ECMO bridge to LTx, which raises medical, ethical as well as resource allocation issues.

## Case presentation

**Fig. 1:**
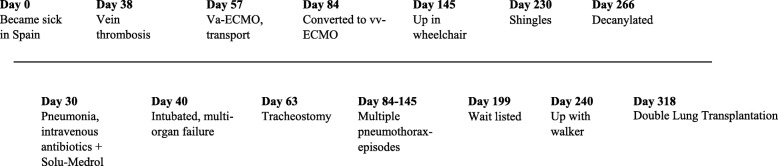
Timeline for the 59-year-old female patient

A 59-year-old woman, previously healthy, developed eye related symptoms, swelling and itching, nightly fever and muscle weakness during vacation in Spain. She had a fever of 40 degrees and received antibiotics. A relapse was treated with intravenous antibiotics and steroids for suspected pneumonia. Anti-nuclear and anti-Sjogrens-syndrome antibodies were positive. She was intubated due to respiratory insufficiency and developed multi-organ failure, thrombocytopenia and bleeding in lungs and stomach. Multiple lung infiltrates on computed tomography scan with progressive hypoxemia resulted in an emergency call to a mobile extra-corporeal membrane oxygenation team, who flew to Spain, initiated veno-arterial ECMO and transported her back to Sweden. She was later converted to veno-venous ECMO and eventually diagnosed with dermatomyositis. Her kidney function was low with a measured glomerular filtration rate of 12 ml/min and she required hemodialysis. She was tracheotomized in order to manage airway secretions. Over time (Fig. 1), her situation stabilized and she was fully awake, mentally adequate, drinking champagne on her wedding anniversary and exercised with bed cycling. She was highly motivated but found to have at least one contraindication for lung transplantation due to chronic renal replacement therapy. Despite multiple assessments after which she was turned down on multidisciplinary board it eventually became unethical not to accept her for lung transplantation listing. After 229 days on ECMO she underwent double LTx. She needed veno-venous ECMO for 2–3 days post-operatively and weaned thereafter. She stayed in the intensive care unit for 33 days, and had an otherwise uneventful albeit long recovery. She was eventually discharged after another 9 days and continued to recover slowly at her referring hospital. After 14 months she was kidney transplanted. She is now clinically stable and lives a normal life.

**Fig. 2:**
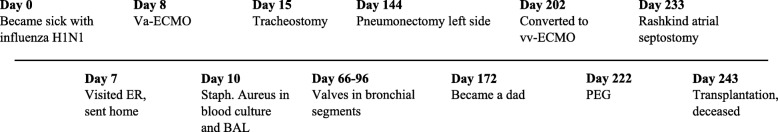
Timeline for the 34-year-old male patient

A 34-year-old man with Diabetes Mellitus contracts influenza A (H1N1) virus and develops a secondary sepsis with PVL-toxin-producing *Staphylococcus aureus*, causing necrotizing pneumonia with destruction of the lungs. Combined respiratory and circulatory failure occurred and he required veno-arterial extra-corporeal membrane oxygenation. He was tracheotomized in order to manage airway secretions. The left lung was completely destroyed and bleeding necessitated finally pneumonectomy. He was awake on ECMO and learns during his hospital stay that his wife was pregnant with their first child. He was turned down for LTx several times, but when he finally stabilized, the decision was changed and he was listed for LTx (Fig. 2). After 281 days on ECMO he received single LTx on the right side. The operation was carried out through sternotomy and was complicated by severe adhesions. It was also noted intraoperatively that fibrotic transformations around the vessels had progressed during waiting time compared to work-up and resulted in great difficulties to identify the anatomic structures. Parts of the lower lobe had to be left in situ, and the patient died of bleeding intra operatively.

## Discussion and conclusion

We have in this case report illustrated the difficulties in managing patients, not initially being on the waiting list for lung transplantation, who end up being stable and awake but requiring continuous extra-corporeal membrane oxygenation treatment, which cannot be weaned due to completely destroyed lungs. Both patients spent > 200 days on ECMO and were initially deemed unacceptable for lung transplantation, but eventually transplanted with different outcome.

Intuitively, the younger patient of the two would have had a better chance of survival, however the older one did not only survive LTx but also a long period with hemodialysis before kidney transplantation was successfully performed. After a similar long duration of ECMO, in aftermath the younger patient was inoperable due to not only severe adhesions but also to a fibrotic chest, likely related to a long-standing state of non-ventilation and multiple thoracotomies. In addition, our surgical approach may be questioned, and thoracotomy with vaECMO from groin would maybe have been a more traditional approach. We perform most of our LTx through sequential thoracotomies, and we opted for this other approach since we believed we would need to go intrapericardially due to vast changes in the hilum.

The older female patient had a very strong and compliant personality making her an ideal candidate for a demanding treatment with only one contraindication to LTx (dialysis), whereas the younger patient with a very difficult pre-transplant course in aftermath should have been declined LTx due to multiple contraindications (dialysis, fibrotic chest, pneumonectomy preventing bilateral LTx). He should have been declined not because of the long ECMO run, and not because of age, but mostly because of chronic fibrotic changes in his lungs and chest, due to long-standing non-ventilation and multiple thoracotomies. Although he was denied LTx on multiple occasions due to infection, bleeding and surgeries, we eventually accepted him for compassionate reasons. We chose sternotomy in order to keep intrapericardial options open, since massive adhesions were expected, and since we already were on mechanical circulatory support and planned to do the case on heart lung machine. Upon transplantation, central anatomy and large vessels were not possible to distinguish among the extensive fibrotic scar tissue, never even before experienced by any of the surgeons of the team, and the patient basically died of intraoperative bleeding.

In general, it has been shown that ECMO bridge-to-lung transplantation is feasible nowadays, however, with somewhat higher risk for mortality and morbidity (1, 2). Even if new technology has made it possible to manage patients > 200 days on extra-corporeal membrane oxygenation without dying, it does not necessarily mean that we should do it. But it is extremely difficult out of an ethical point of view to turn down an otherwise intact individual, who has no other option than death upon weaning.

We conclude, that even if decisions regarding who should be selected for extra-corporeal membrane oxygenation treatment are difficult, the next patient similar to our young man with a long-standing non-ventilated chest with a non-compliant lung will in our program be turned down for LTx. However, the positive outcome of the older woman well illustrates that these decisions, both out of medical and ethical perspectives, are not without difficulties and that maybe one contraindication can be accepted upon bridge to LTx.

## Data Availability

Not applicable.

## Abbreviations

ECMO: Extra-corporeal membrane oxygenation
LTx: Lung transplantation
